# A Cruise to the Canaries

**Published:** 1911-04-01

**Authors:** 


### The Practitioner's Relaxations.

### A CRUISE TO THE CANARIES.

As a result of the notice which appeared in The Hospital of January 14, concerning the three weeks' trip to the Canaries, arranged by Yeoward Brothers, of 27 to 29 Stanley Street, Liverpool, for the extraordinarily cheap sum of twelve guineas, one of our readers sailed on the "Aguila" on February 11. The following is an edited account of his experience: I notice that almost every previous writer on the practitioner's relaxations has assumed that doctors are never happy unless they are indulging in the collector's passion in some form. From birds' eggs to old lace or furniture, the acquisition of some rarity has generally been recommended. Now these are all rich men's pleasures, requiring for their pursuit a continual expenditure. No apology therefore is needed for recommending a short cruise, for this relaxation is one of the most popular among medical men, as the number of applicants for the post of ship's doctor proves, and the sum of money it is going to cost is settled beforehand at a very low figure.

### The Voyage Out.

One arrived at the Coburg Dock, Liverpool, to find a ship, bigger than one had thought from her photographs, of 2,500 tons, capable of carrying some seventy passengers. The doctor who really wants a rest should certainly go in the spring or the autumn, for not only is the weather in England most unpleasant then, but there is also less chance of the boat being crowded. The first place of call is Lisbon, which is reached in fine weather in about five days. One has just found one's sea legs when the cheering sight of land is greeted after the passage of the Bay of Biscay, only the western-most side of it, by the way, being crossed. Almost the first thing which strikes the passenger approaching Lisbon by sea is the site of the Palace from which King Manoel made his escape to the royal yacht. Undoubtedly the finest thing to be seen at Lisbon is the harbour, which is formed by the great basin into which the Tagus opens near its mouth, and which appears large enough to shelter all the navies of Europe. The city itself was shattered in an earthquake some hundred years ago, and this no doubt accounts for the modernity of the buildings. Only one old church was readily to be seen, and an excursion must be made to the suburbs for any architecture worth looking at.

### Excursions at Lisbon.

Yeoward Brothers send their agent on board, and it is possible to be conducted by him round the city, to the bull-ring, to the Black Horse Square, where the King was assassinated, and so on. Besides this there are what may be called regular excursions, to Cintra, where the above-mentioned palace and that belonging to the Dowager Queen may be seen, to the well-known Montserrat Gardens on the way, or to Mont Estoril, a fashionable seaside resort with a casino, or again to Belem, where is the famous monastic church. The former are excursions for the pleasure-seeker, including as they do a short journey by train, a drive, lunch in an hotel, sight-seeing, and the return journey; the latter, Belem, is for the man of discrimination who has taken pleasure in church architecture at home. Belem, now tenantless of monks, the refuge of military orphans under Government supervision, has lost by the revolution nothing of its ancient glory. The beautiful cloisters are still unimpaired, and the great church still provides a superb canopy for the famous tombs, where Camoëns and Herculano, Vasco da Gama, and many kings lie buried. The Moorish influence is strongly seen in the intricacy and often formal pattern of the carving, which is to be found luxuriantly about the church. In the above ways the two or three days at Lisbon may be spent.

### Life on the Boat.

These excursions have been emphasised because they form an important feature of the trip, and are probably the most enjoyable part of it to those people who have not come for the delight of sea-air and sunshine alone. For these however an important fact about the "Aguila" should be mentioned—the very large area of her promenade deck, which has a proportion unusually great for a ship of her size. Life on board begins for most passengers with breakfast, which is served at 8.30, and as there is always little else to do but eat on a boat, it is worth while mentioning a sample of the food offered for breakfast. Porridge, soused fresh fish (though the experienced traveller generally avoids fish at sea in spite of the modern devices for refrigeration), Irish stew, American dry hash, bacon (eggs to order), braised beef, perhaps boar's head—these, with jam, marmalade, and golden syrup, give a fair idea of the choice of food. Often apples or bananas were on the table, and of course either tea or coffee could be had. After leaving Lisbon the "Aguila" calls normally at Madeira, but on this trip that was postponed till after the Canaries had been visited, Madeira on the way out not having been declared a clean port. Getting on board again after Lisbon, with most chances of rough weather passed, the sun growing every day more pleasantly hot, the normal pleasures of a boat begin again. These are not to be found so much in the various deck games that many generations of sea travellers have invented as in the two interests of the other passengers and of meals. Both inevitably provide the principal distractions of sea life. The passengers fall naturally into types: there is probably the young consumptive man or woman going out to the dry climate of Teneriffe for his or her health; there is the rich tourist from the town; there is the professional man, perhaps with his family, who has escaped from work at a time when most professional men are busy; there are the young ladies who are every one's and no one's. Some of these will probably be found, and their combination is sure to produce some form of amusement. Every ship's saloon contains a crowd, remember, which has no other common interest but a desire to leave the same country, and this is bound to provide some entertaining contrasts.

### The Islands and the Peak.

Las Palmas is the next place of call. It is the capital of Grand Canary, with a harbour that is the envious rival of Madeira, and which has benefited by her misfortunes. It is also the place where the open work on linen that is done by the natives may first be bought. Cigarettes are also obtainable at impossibly low prices, and the factory where they are made may be seen. The great excursion here is the drive up to Monte, where are to be found some very pretty gardens with oranges growing in them belonging to the hotels, in contrast to the wild appearance of the hills around them. These hills, if I remember, have little cultivation but bananas, and they grow near the water, on their lower slopes. Atalaya, the cave village, typical of the houses where the ancient Guanches used to live, is included in the day's programme. The only human trace of this race is to be found in the blue eyes that a few of the children in this district still surprise one by possessing. Native pottery is made here also. By night the steamer generally goes on to Tenerife, and the next day can be spent at Santa Cruz. A drive here can be made to Laguna, or indeed, if one is sure rough weather will not prevent the ship calling at Orotava on the other side, right across the island. Orotava seemed to the present writer the most beautiful of the island towns. Here also are the Yeoward Brothers' banana plantations, and among them lunch is served for those who like the drive to them. As the ship lies outside the rocks of Orotava the Peak is seen standing finely with snow on its summit at what appears to be just over the town. Its height is curtailed by a range of hills which hide its base, but, especially as the ship is leaving, it looks very fine, and a land view of it can be obtained from Tacoronte, which is beyond Laguna. In the summer the Peak is ascended often by passengers. The whole of the Canary Islands can be seen from the top. Anyone who wishes to go, as the excursion book reminds one, must give notice before leaving Las Palmas, so that guides and mules can be hired. The cost varies with the number of the party, but I forget the exact sum.

### Madeira.

On the return journey Santa Cruz and Las Palmas are visited again, cargoes being carried between the islands, and this particular cruise ended with what is the finest sight of the whole tour, I mean Madeira. Funchal is the port, and the vegetation in itself makes walking delightful. Everyone goes by the funicular to the Mount, which is 2,000 ft. up, lunches there, and toboggans down the cobbled road at whatever speed the not very athletic men who hold the guiding strings choose to venture. Everything is offered for sale in Madeira, but everything, including the cost of living, is expensive. It remains, however, more luxuriant than anything to be found in the Canaries; everything seems to grow, as there is plenty of sunshine and more rain than in the islands.

The "Aguila" had the distinction of being the first ship to land passengers after the cholera, and the passengers were welcomed by the inhabitants with fireworks and other signs that the Englishman with money to spend and on his holiday is appreciated sincerely.

### The Accommodation Offered.

Enough has been said perhaps to show that a spring or summer cruise to the Canaries can be offered as a first-rate relaxation to the practitioner. It offers three weeks of sunshine, abundance of fresh air, the opportunity to see, or, if preferred, easily to refrain from seeing, many places, Lisbon, the famous Madeira, or the Canaries, which possess, perhaps, the finest climate in the world within equally easy reach of England. The boat itself has all the comforts which belong to modern ships which aim at convenience rather than size or speed. It is big enough to make rough weather no severe anxiety, to give reasonably-sized cabins, and yet to avoid the continual vibration of a racing screw by which more pretentious boats are often spoilt. In conclusion, let us add a typical example of the menu for dinner. Soup, salmon from tins, sweetbreads, Hungarian ghoulash, roast beef, savoury hot-pot, corned beef, curry, prunes and rice, cheese, bananas, oranges, or almonds and raisins, and coffee, which is served in the drawing-room, the smoking-room, or on deck. The saloon and the two sitting-rooms just mentioned provide a refuge on the rare occasions when the weather has to be feared. At least one learnt that the trip which started on February 11 was the fourth in succession which had been completely fine. This three weeks' cruise is one that is likely to attract medical men from its frequency, its cheapness, and the healthy conditions which it provides. The "Aguila" sails again on April 8, and one of the fleet every Saturday.

